# Subsurface Damage in Polishing Process of Silicon Carbide Ceramic

**DOI:** 10.3390/ma11040506

**Published:** 2018-03-27

**Authors:** Yan Gu, Wenhui Zhu, Jieqiong Lin, Mingming Lu, Mingshuo Kang

**Affiliations:** School of Mechatronic Engineering, Changchun University of Technology, Changchun 130012, China; guyan@ccut.edu.cn (Y.G.); 201501036@stu.ccut.edu.cn (W.Z.); lumm@ccut.edu.cn (M.L.); 20151768@stu.ccut.edu.cn (M.K.)

**Keywords:** SiC ceramic, subsurface damage, polishing process, finite element simulation

## Abstract

Subsurface damage (SSD) in the polishing process of silicon carbide (SiC) ceramic presents one of the most significant challenges for practical applications. In this study, the theoretical models of SSD depth are established on the basis of the material removal mechanism and indentation fracture mechanics in the SiC ceramic polishing process. In addition, the three-dimensional (3D) models of single grit polishing are also developed by using the finite element simulation; thereby, the dynamic effects of different process parameters on SSD depth are analyzed. The results demonstrate that the material removal was mainly in brittle mode when the cutting depth was larger than the critical depth of the brittle material. The SSD depth increased as the polishing depth and abrasive grain size increased, and decreased with respect to the increase in polishing speed. The experimental results suggested a good agreement with the theoretical simulation results in terms of SSD depth as a function of polishing depth, spindle speed, and abrasive grain size. This study provides a mechanistic insight into the dependence of SSD on key operational parameters in the polishing process of SiC ceramic.

## 1. Introduction

A new generation of space-to-ground optical information collection systems requires high ground resolution and a large coverage width, so as to make the optical systems constantly highly lightweight, as well as have a large diameter and an off-axis. This also inspires the development of optical remote sensing camera key components that are made of a high-quality light optical reflector material [1]. Silicon carbide (SiC) ceramic represents a kind of ideal space mirror material by virtue of its high hardness and strength, excellent chemical stability, and good wear resistance [2,3]. However, there remain challenges associated with the inherent properties of SiC, such as its high brittleness and low fracture toughness, which may lead to subsurface microcracks, dislocation, deformation, and residual stresses. This may heavily degrade the mechanical strength and fatigue properties, as well as its adoption of surface integrity [4]. Computer-controlled optical surfacing has been widely used in precision machining of hard and brittle materials. However, the precision polishing process of silicon carbide ceramic is limited in many aspects; its process is simple, and there may be polishing subsurface damage (SSD).

At present, it is common practice to investigate the polishing mechanism by establishing the models of material removal depth based on the Preston equation according to dh=kpvdt [5,6]. However, the constant *k* (i.e., factor influence coefficient) is an empirical parameter that needs correction depending on the results of time and labor-intensive experiments. In addition, it is also noticed that the Preston equation is a macroscopic model, i.e., it cannot reflect the effect of the lack of abrasive characteristics on the depth of removal. From the microscopic point of view, a number of studies described the polishing process, focusing on the complicated interaction between abrasive grains and the workpiece. For example, Wang et al. established equations describing the relationship between pressure and cutting depth, taking into account abrasive particle size as an important parameter of removing depth; they also gave mathematical models for linear removal strength [7]. Qi et al. considered the impact of the characteristics of abrasive grains on removal depth and linear removal strength when establishing the microscopic models of material removal depth during polishing [8]. These studies, from the perspective of abrasion, will be helpful for better understanding the effect of material removal depth on SSD.

Recently, many theoretical analyses, numerical simulation methods, and experimental methods have been developed for investigating the material removal mechanism and SSD in the process of brittle material processing. In theory, Wang et al. applied the indentation fracture mechanics of brittle materials to establish the predictive models of cutting force and the SSD depth of K9 optical glass during ultrasonic rotary surface machining [9]. Liu et al. reported the mathematical relationship between the depth of subsurface cracks and the processing parameters during the silicon wafer cutting process, according to the principle of indentation fracture mechanics [10]. Esmaeilzare et al. used a cup diamond wheel in the grinding process of the specimens made of Zerodur^®^ glass–ceramic to investigate the influences of grinding parameters on the SSD and surface roughness (SR), and established a statistical model for the prediction of SR and SSD depth [11]. Chen et al. also proposed a new model to analyze the relationship between SR and SSD depth on the basis of grinding kinematics analysis and the indentation fracture mechanics of brittle materials that took the wheel spindle vibration into account [12]. Recently, the simulation technology of hard and brittle materials in precision machining has been applied more and more in the research of SSD. For example, Zhu et al. utilized single-grit simulation to investigate the initiation and propagation of individual cracks under controllable maximum undeformed chip thickness in SiC grinding [13]. Liu et al. conducted single-grit engagement simulations to investigate the effects of the high speed on the surface/subsurface cracks and grinding forces [14]. Komanduri et al. carried out a molecular dynamics (MD) simulation of nanometric cutting with tools of different edge radii relative to the depth of cut, and investigated the variations of cutting forces, thrust forces, the force ratio, the specific energy, and the subsurface deformation with the tool geometry and depths of cut; their results found that they all have a significant influence [15]. Dai et al. employed three-dimensional molecular dynamics (MD) simulations to investigate the effect of tool geometry on subsurface damage and material removal in nanometric cutting single-crystal silicon [16]. Li et al. also used MD simulations to investigate the effects of grinding speed on grinding damage and grinding surface integrity through the analysis of chips, dislocation motions, and phase transition in a single-crystal silicon grinding process [17]. In experimental testing, Lucca et al. employed a variety of techniques to assess the surface alterations that occur as a result of processing, and they presented examples of the processing of metals, ceramics, and glasses in order to assess the nature and extent of surface alterations [18]. Agarwal et al. studied the grinding characteristics, surface integrity, and material removal mechanisms in diamond wheel-ground SiC surfaces, and the study combined surface roughness (SR), surface damage, and subsurface damage with grinding parameters, which provided valuable insights for the material removal mechanism and the dependence of grinding damage on grinding conditions [19]. Jiang et al. carried out a series of K9 glass precision grinding tests, and studied the influence of grinding parameters on SSD. The experimental results show that the material removal rate was closely related to SSD [20]. Blaineau et al. obtained the relationship between SSD depth and grinding force by comparing experimental results with discrete element method simulation results [21]. Wang et al. used the method of theoretical analysis, numerical simulation, and experimental testing to analyze the SSD during the high-speed grinding of brittle materials [22]. However, theoretical studies on the SSD of hard brittle materials in the polishing process have been reported with limited success, which is likely because the majority of existing studies rarely take systematic theoretical models for SSD and three-dimensional (3D) dynamic analysis into consideration. Thus, it will be highly desirable to develop the methods based on finite element simulation for more accurately predicting and controlling the SSD depth during the polishing of hard and brittle materials.

The main purpose of this study is to establish the theoretical models of SSD by taking into account the removal mechanism for the SiC ceramic polishing process. In order to obtain the relationship between SSD and processing parameters (polishing depth, tool speed, and abrasive grain size) on a quantitative basis, the 3D finite element simulation is performed to simulate the process of single-grit polishing SiC ceramic. A series of experiments were carried out to validate the theoretical and simulation results.

## 2. Theoretical Analysis

### 2.1. SSD Models

**Figure 1:**
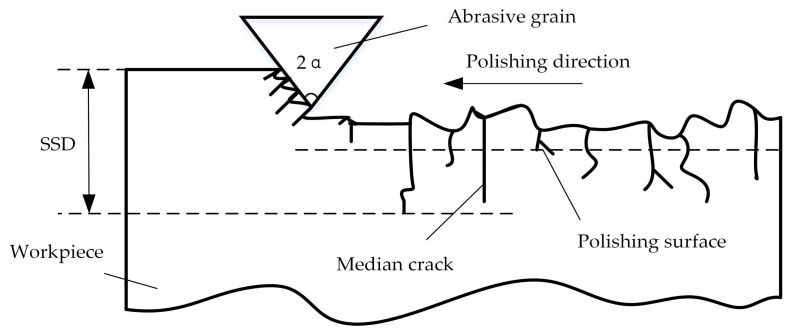
Scratch process of single abrasive grain.

**Figure 2:**
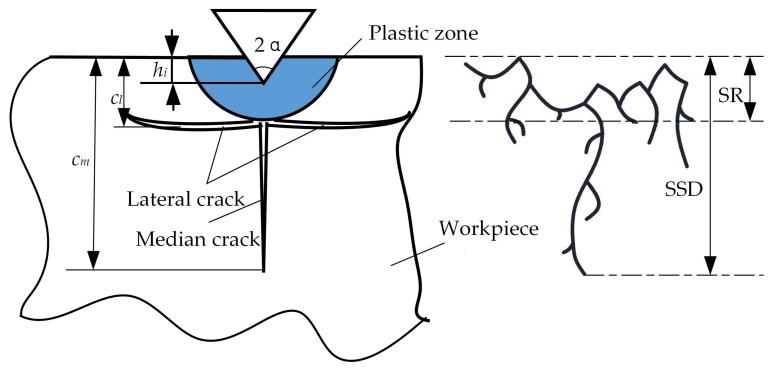
Subsurface damage model.

The contact polishing of hard and brittle materials can be simplified in an abstract way into a single-grit scratch process [23], which can be schematically illustrated in Figure 1. Figure 2 shows the lateral cracks in association with material removal and surface formation. The median cracks causes SSD, and thus, the degradation of strength of the material [24]. $c_{m}$ is the median crack length at the bottom of the plastic zone under peak load or critical conditions in the scratch process; $c_{l}$ is the transverse crack depth from the bottom of the plastic deformation zone; $h_{i}$ is the depth of penetration of the abrasive particles and the workpiece; and *α* is abrasive grain apex angle.

The median crack can be classified as typical SSD, as it is perpendicular to the polishing surface, and extends to the machined plane. According to the indentation fracture mechanics, the depth of median crack *c_m_* can be correlated to material properties, the geometry of the abrasive grain, and penetration depth [25] according to:

(1) $$c_{m}=0.206\frac{{\left(E\cdot H_{s}\right)}^{1/3}}{{\left(K_{c}\cdot \beta \right)}^{2/3}}{\left(\cot\alpha \right)}^{4/9}{\left(\tan\alpha \right)}^{4/3}\cdot {\left(h_{i}\right)}^{4/3},$$

where *H_s_* (GPa) is the scratch hardness, *K_c_* (3.5 MPa⋅m^(1/2)^) is the fracture toughness, and *β* (0.363) is the material parameter determined by elastic recovery.

The SSD depth can be derived from the geometric relationship between the abrasive and the workpiece, which is shown in Figure 2 as:

(2) $$\mathrm{SSD}=\max(c_{m}),$$

### 2.2. Models of Single-Grit Penetration Depth

#### 2.2.1. The Height Distribution of ABRASIVE Protrusion

Polishing is a complex material removal process involving rubbing, scratching, plowing, and cutting, which is necessarily analyzed by using probability statistics. The abrasive grain sizes are determined in terms of the value of grit (generally indicated by *M*) [26]. The maximum diameter $d_{\max}$ and minimum diameter $d_{\min}$ of the abrasive grains can be determined when the grit is known. The mean diameter $d_{m}$ is defined by $d_{m}=\left(d_{\max}+d_{\min}\right)/2$. The number of the abrasive grains per unit area can be calculated as:

(3) $$N=\frac{6V_{g}}{{{\pi d}_{m}}^{2}},$$

where $V_{g}$ (%) is the grain ratio, and the structure number *S* stands for the volume ratio of the grains in the whole element volume. The relationship between *S* and the volume ratio is $V_{g}=2\left(31-S\right)$, for instance: $V_{g}V_{g}$ is 62 and 44, when *S* = 0 and *S* = 9, respectively [27].

Massive experiments have shown that both $d_{\max}$ and $d_{m}$ are very close to the maximum and the average heights of the abrasive protrusion, respectively [7]. Hence, the distribution of the abrasive grain protrusion heights of the polishing tool surface accords well with Gaussian distribution as:

(4) $$f\left(h\right)=\frac{1}{\sqrt{2\pi }\sigma }e^{\frac{-{\left(h-u\right)}^{2}}{2\sigma^{2}}},$$

where *h* is the height of the abrasive protrusion, the mean value $u=d_{m}$, and the standard deviation $\sigma =\left(d_{\max}-d_{m}\right)/3$. Values of $d_{m}$, $d_{\max}$, and *σ* for different grain sizes are shown in Table 1.

**Figure 3:**
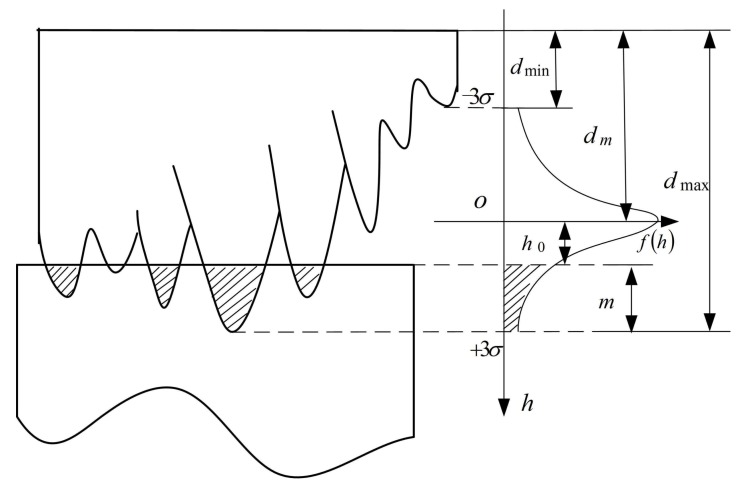
Schematic illustration of the abrasive grain heights distribution.

Figure 3 illustrates a schematic diagram of the grain protrusion heights distribution in the abrasive tool surface. The origin of the coordinate system is fixed on the horizontal position of $d_{m}$, and $h_{0}$ is the vertical distance from the origin 0 and workpiece surface. It is based on the geometric relationship $h_{0}=3\sigma -m$, where *m* is the indentation depth m∈[0,6σ]. Considering that $\int_{-3\sigma }^{3\sigma }f\left(h\right)dh=0.9973$, the probability density function of the abrasive grain protrusion heights distribution can be written as:

(5) $$f\left(h\right)=\left\{ \begin{array}{ll} \frac{1}{\sqrt{2\pi }\sigma }e^{\frac{-h^{2}}{2\sigma^{2}}} & \left|h\right|\leq 3\sigma \\ 0 & \left|h\right|>3\sigma \end{array}\right.,$$

#### 2.2.2. The Number of Effective Grains

Through the above analysis, we can conclude that not all of the grains are involved in the polishing process. The material removal process will happen only when the protrusion height is bigger than $h_{0}$. From Equation (5), the particle distribution probability of the abrasive grain height higher than $h_{0}$ in unit area is obtained:

(6) $$P\left\{h_{0}<h<3\sigma \right\}=\int_{h_{0}}^{3\sigma }f\left(h\right)dh,$$

Combining Equations (3) and (6) gives the number of effective grains in the following form:

(7) $$N_{r}=\frac{6V_{g}}{\pi {d_{m}}^{2}}\int_{h_{0}}^{3\sigma }f\left(h\right)dh,$$

#### 2.2.3. The Depth of a Single Abrasive Grain

The contact deformation between diamond abrasive grains and the SiC workpiece can be classified as the plastic deformation [28]. According to the theory of elastic mechanics, the plastic contact force between a single abrasive grain and workpiece can be described as:

(8) $$F_{a}=2{h_{i}}^{2}H_{w},$$

where $H_{w}$ is the hardness of the workpiece, and $h_{i}$ is the penetration depth of the abrasive particles.

According to the principle of mechanical equilibrium, the contact force between the polishing tool and the workpiece (the contact force between the effective abrasive particles and the workpiece) is equal to the polishing force:

(9) $$F=N_{r}F_{a}=2{h_{i}}^{2}H_{w}\frac{6V_{g}}{\pi {d_{m}}^{2}}\int_{h_{0}}^{3\sigma }f\left(h\right)dh,$$

Therefore, the penetration of a single abrasive grain into the workpiece is obtained as:

(10) $$h_{i}=\sqrt{\frac{F}{2H_{w}\frac{6V_{g}}{\pi {d_{m}}^{2}}\int_{h_{0}}^{3\sigma }f\left(h\right)dh}}=\sqrt{\frac{\pi F{d_{m}}^{2}}{12H_{w}V_{g}\int_{h_{0}}^{3\sigma }f\left(h\right)dh}},$$

Substituting Equation (10) for Equation (2) gives that:

(11) $$\begin{array}{ll} SSD & =\max\left[0.206\frac{{\left(E\cdot H_{s}\right)}^{1/3}}{{\left(K_{c}\cdot \beta \right)}^{2/3}}{\left(\cot\alpha \right)}^{4/9}{\left(\tan\alpha \right)}^{4/3}\cdot {\left(\frac{\pi F{d_{m}}^{2}}{12H_{w}\int_{h_{0}}^{3\sigma }f\left(h\right)dh}\right)}^{2/3}\right]\\ & =\max\left[\lambda \cdot {\left(\frac{\pi F{d_{m}}^{2}}{12H_{w}V_{g}\int_{h_{0}}^{3\sigma }f\left(h\right)dh}\right)}^{2/3}\right] \end{array},$$

where *λ* is the proportional coefficient related to material properties and head geometry, whose value can be calculated by:

(12) $$\lambda =0.206\frac{{\left(E\cdot H_{s}\right)}^{1/3}}{{\left(K_{c}\cdot \beta \right)}^{2/3}}{\left(\cot\alpha \right)}^{4/9}{\left(\tan\alpha \right)}^{4/3},$$

The material characteristic parameters of SiC ceramics are shown in Table 2.

### 2.3. Analysis for Dynamic Parameters

**Figure 4:**
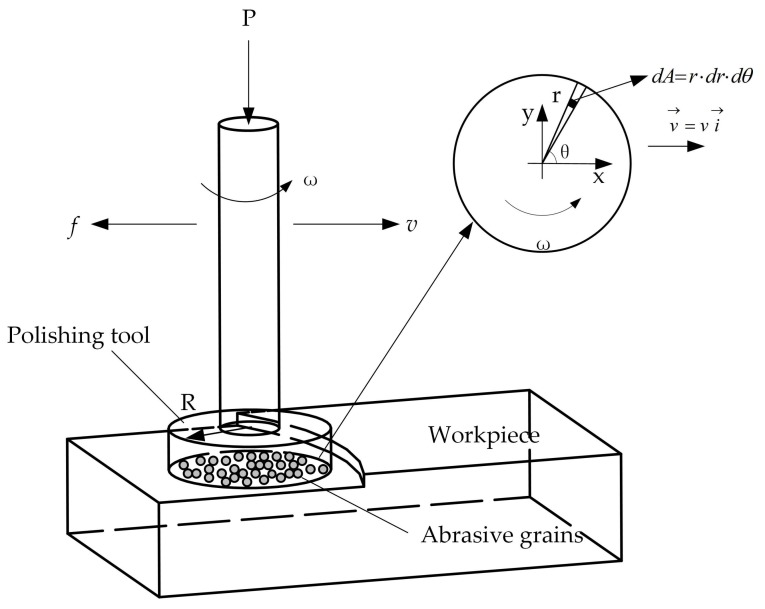
Schematic diagram of the motion between the polishing tool and the workpiece.

Equation (9) shows that the depth of a single abrasive cut into the workpiece is related to the particle size and contact force of the abrasive. Figure 4 schematically illustrates the diagram of the motion between the polishing head and the workpiece. The force as a function of velocity at any point on the polishing head can be calculated by a kinematic geometric relationship. As shown in Figure 4, the tangential unit vector can be obtained at the radius *r* as:

(13) $$\hat{\tau }=-\sin\theta \vec{i}+\cos\theta \vec{j},$$

The linear velocity $\vec{v}$ at *r* can be written as:

(14) $$\vec{v_{r}}=\omega r\cdot \hat{\tau }=-\omega r\sin\theta \vec{i}+\omega r\cos\theta \vec{j},$$

Since the feed rate of the polishing head is $\vec{v}=v\vec{i}$, the absolute rate $\vec{v_{a}}$ at radius *r* should be given as:

(15) $$\vec{v_{a}}=\vec{v}+\vec{v_{r}}=\left(v-\omega r\sin\theta \right)\vec{i}+\omega r\cos\theta \vec{j},$$

Therefore, the unit vector at radius *r* can be given as:

(16) $${\hat{v}}_{a}=\frac{\vec{v_{a}}}{v_{a}}=\frac{1}{\sqrt{v^{2}+\omega^{2}r^{2}-2v\omega r\sin\theta }}\left[\left(v-\omega r\sin\theta \right)\vec{i}+\omega r\cos\theta \vec{j}\right],$$

The normal stress on the disk surface is $\sigma =P/A=P/\left(\pi R^{2}\right)$. The frictional force at dA is df=μσdA, and the vector form is $d\vec{f}=-{\hat{v}}_{a}\cdot df$. Thus, the friction force of the disc can be expressed as:

(17) $$\vec{f}=\int d\vec{f}=-\iint \frac{\left(v-\omega r\sin\theta \right)\vec{i}+\omega r\cos\theta \vec{j}}{\sqrt{v^{2}+\omega^{2}r^{2}-2v\omega r\sin\theta }}\cdot \frac{\mu P}{\pi R^{2}}\cdot r\cdot dr\cdot d\theta ,$$

Clearly visible is the resultant force of zero on the disk in the y-direction. In order to simplify Equation (17), we consider $1/\sqrt{v^{2}+\omega^{2}r^{2}-2v\omega r\sin\theta }$ for McLaughlin expansion at v=0:

(18) $$\begin{array}{ll} \frac{1}{\sqrt{v^{2}+\omega^{2}r^{2}-2v\omega r\sin\theta }} & =\frac{1}{\sqrt{v^{2}+\omega^{2}r^{2}-2v\omega r\sin\theta }}{|}_{v=0}-\frac{\omega r\sin\theta -v}{{\left(v^{2}+\omega^{2}r^{2}-2v\omega r\sin\theta \right)}^{3/2}}{|}_{v=0}\cdot v+ο\left(v\right)\\ & =\frac{1}{\omega r}+\frac{\sin\theta }{\omega^{2}r^{2}}v+ο\left(v\right) \end{array},$$

where ο(v) is the infinitesimal of higher order.

Substituting Equation (18) for Equation (17) gives the friction of the disk as:

(19) $$\vec{f}=-\frac{\mu Pv}{\omega R}\vec{i},$$

As can be seen from the geometric relations in Figure 4, the force between the polishing head and the workpiece can be written as a function of friction and pressure as:

(20) $$F=\sqrt{f^{2}+P^{2}}=P\cdot \sqrt{1+\frac{\mu^{2}v^{2}}{\omega^{2}R^{2}}},$$

Substituting Equation (20) to Equation (11) gives:

(21) $$SSD=\max\left[\lambda \cdot {\left(\frac{\pi Pd_{m}^{2}}{12H_{w}V_{g}\int_{h_{0}}^{3\sigma }f\left(h\right)dh}\right)}^{2/3}\cdot {\left(1+\frac{\mu^{2}v^{2}}{\omega^{2}R^{2}}\right)}^{1/3}\right],$$

### 2.4. Analysis for SSD Depth Models

In brittle material processing, if the material is removed to the critical value of brittle fracture, the brittle mode (transverse crack or intermediate crack) occurs. The critical cutting thickness $h_{c}$ can be written as [29]:

(22) $$h_{c}=0.15\left(\frac{E}{H_{w}}\right){\left(\frac{K_{c}}{H_{w}}\right)}^{2},$$

For $h_{i}>h_{c}$, the fragile mode takes place in the SiC polishing process, and thus, the value of $h_{c}$ can be calculated as 0.0328 μm. The $h_{i}$ value can be estimated as 0.5878 μm according to Equation (10), where P=10 *N* and $d_{m}=8.8$ μm. In this case, the material removal is in the brittle mode, i.e., $h_{i}>h_{c}$.

In the brittle mode polishing process, Equation (21) can be used for initially determining the processing parameters. The formula gives the relationship between the parameters of the wheel speed *ω*, the feed speed *v*, the apex angle of abrasive grain *α*, and the applied load *F* and SSD depth. With the increasing of feed speed, particle angle and load, the SSD depth increases; however, with the increasing of polishing speed, the SSD depth decreases. In addition, there is a certain proportion of the external load on the surface of the workpiece in the polishing process, and the polishing depth in the experiment.

## 3. Numerical Simulation

### 3.1. Constitutive Models

The polishing process of the SiC ceramic produces a new surface as a result of brittle fracture. Thus, it creates an easy way to fabricate subsurface microcracks, dislocations, deformation, and residual stress damage. Correspondingly, it is also necessary to establish a fracture failure model to predict subsurface damage. In this study, the JH-2 constitutive model was used to describe the fracture damage of ceramic polishing [14,30] as:

(23) $$\sigma =\left(1+C\ln\overset{\cdot }{\varepsilon }\right)\sigma_{HEL}\left[A{\left(\frac{P+T}{P_{HEL}}\right)}^{N}-D\left(A{\left(\frac{P+T}{P_{HEL}}\right)}^{N}-B{\left(\frac{P}{P_{HEL}}\right)}^{M}\right)\right],$$

where *σ* (GPa) is the actual equivalent stress of SiC, $\sigma_{HFL}$ (GPa) is the equivalent stress at Hugoniot, *P* is the hydrostatic pressure, and $P_{HEL}$ (GPa) is the hydrostatic pressure at Hugoniot. *A*, *B*, *C*, *M*, and *N* denote the predetermined parameters of the material. *D* is the material damage coefficient, and *T* (GPa) is the maximum hydrostatic tensile strength.

The hydrostatic pressure of the intact material can be given by [29]:

(24) $$P=\{\begin{array}{ll} K_{1}\mu +K_{2}\mu^{2}+K_{3}\mu^{3}, & \;if\;\mu \geq 0\\ K_{1}\mu , & if\;\mu \leq 0 \end{array},$$

where $\mu =\rho /\rho_{0}-1$, and $K_{1}$, $K_{2}$, and $K_{3}$ are the constants.

When the brittle material begins to damage (*D* > 0), there will be a volume expansion that equals the increase in ΔP. Thus, the hydrostatic pressure to produce damage can be written as [30]:

(25) $${P=K}_{1}\mu +K_{2}\mu^{2}+K_{3}\mu^{3}+\Delta P,$$

Based on the perspective of energy loss, the pressure increment is given by [13]:

(26) $${\Delta P}_{t+\Delta t}=-K_{1}\mu_{t+\Delta t}+\sqrt{{\left(K_{1}\mu_{t+\Delta t}+{\Delta P}_{t}\right)}^{2}+2\beta K_{1}\Delta U},$$

where *β* (0≤β≤1) is the conversion factor of elasticity loss energy and hydrostatic pressure potential, and ΔU (GPa) is the internal energy increment.

Geometrical and physical separation constitute the two main criteria for the separation of the debris and the workpiece. The separation criterion of the JH-2 constitutive equation belongs to the physical separation, which is suitable for characterizing the failure of brittle materials. The material failure parameter *ω* is defined as [31]:

(27) $$\omega =\sum \frac{\Delta {\bar{\varepsilon }}^{pl}}{{\bar{\varepsilon }}_{f}^{pl}\left(P\right)},$$

where $\Delta {\bar{\varepsilon }}^{pl}$ is the increment of the equivalent plastic strain, and ${\bar{\varepsilon }}_{f}^{pl}\left(P\right)$ is the failure strain under the pressure *P*, that is [31]:

(28) $${\bar{\varepsilon }}_{f}^{pl}\left(P\right)=D_{1}{\left(P^{*}+T^{*}\right)}^{D_{2}},{\bar{\varepsilon }}_{f,\min}^{pl}\leq {\bar{\varepsilon }}_{f}^{pl}\leq {\bar{\varepsilon }}_{f,\max}^{pl},$$

where $D_{1}$ and $D_{2}$ are the model constants, and D=ω when the JH-2 model is employed. Supposing that the brittle material undergoes failure (*D* > 1); then, the failure element is removed from the mesh to achieve debris separation.

Table 3 gives the typical constants for the constitutive and failure models, which are determined through a series of experiments.

### 3.2. Simulation Methods

In this study, 3D finite element models are developed using the finite element software ABAQUS/Explicit (Abaqus Inc., Providence, RI, USA). The JH-2 ceramic model is imported by custom materials, which can be used for discussing the relationship among the process parameters and stress distribution, surface morphology, surface, and SSD. In the realistic polishing process, the material removal is produced by the interaction of a large number of random particles distributed on the polishing head and the workpiece. In order to simplify the analysis, the single-grit simulation is used to investigate the subsurface cracks during SiC polishing.

**Figure 5:**
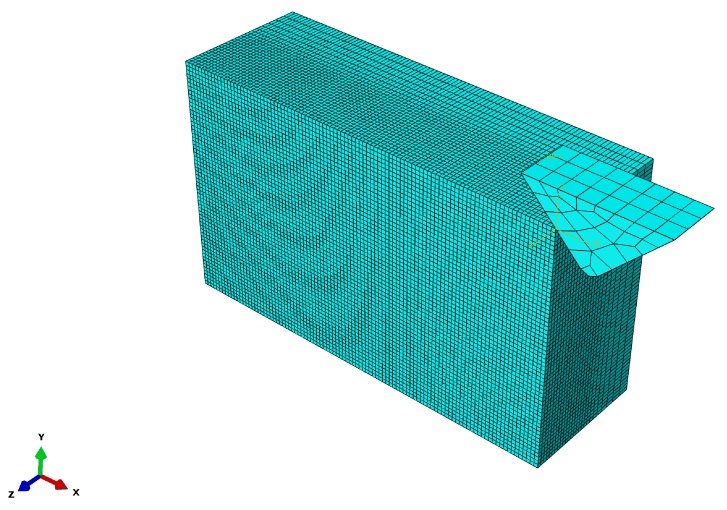
The geometric structure of the three-dimensional (3D) finite element model.

A 3D model is utilized to visualize the initiation and propagation of transverse and median cracks (Figure 5). The size of the workpiece is set 30 μm × 10 μm× 20 μm, which is discretized with a mesh consisting of C3D8R elements. To maximize the calculation efficiency, the model is triturated, and the element is divided densely in the cutting path but has low density in the other regions. The grid encryption area is larger than the contact area between the abrasive particles and the workpiece, so as to prevent the abrasive particles and larger grid contact simulation deviation. The bottom of workpiece is fully constrained, and the boundary condition of abrasive speed is given. The tangential and normal friction on the abrasive–workpiece interface should be considered, which give the friction coefficient of 0.3. In dynamic analysis, a small number of units with small size control the steady time increment. To make the computation more time and cost-effective, the method of mass scaling is adopted. The scaling factor is set as 30. In the simulation process, we only consider the machining effect whilst neglecting the chemical action, the temperature effect of the polishing fluid, and the coupling effect between the abrasive particles. A diamond polishing tool with a diameter of 10 mm is used in the experiment, and the polishing speeds are represented by the maximum linear velocity of the polishing tool. The polishing simulation parameters are shown in Table 4.

### 3.3. Simulation Results and Discussion

#### 3.3.1. Analysis for Single-Grit Polishing Process

**Figure 6:**
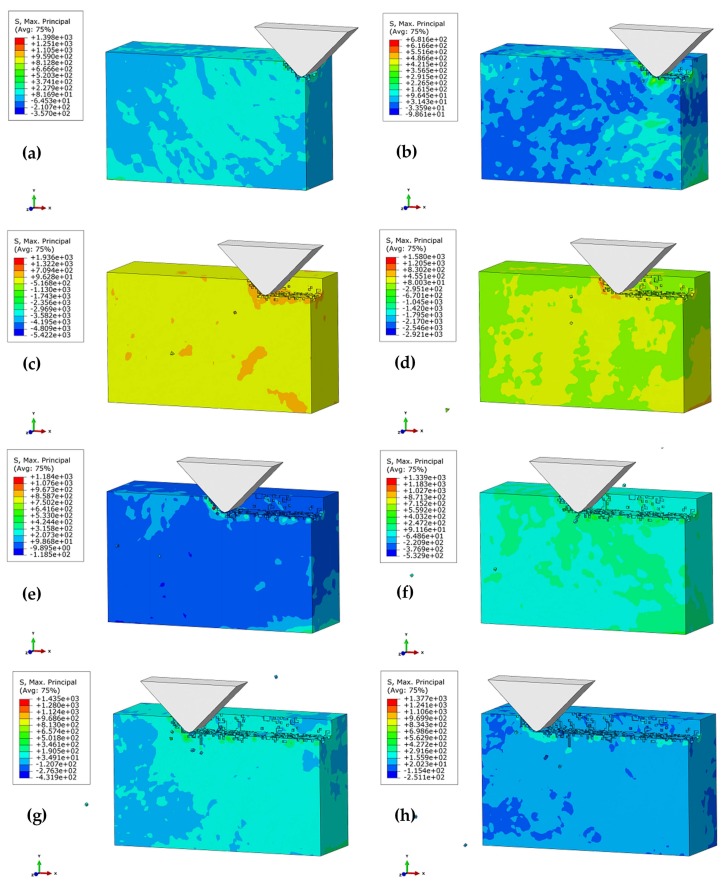
Brittle removal process of the silicon carbide polishing: (**a**) *t* = 2.63 × 10^−6^ s; (**b**) *t* = 5.25 × 10^−6^ s; (**c**) *t* = 7.88 × 10^−6^ s; (**d**) *t* = 1.04 × 10^−5^ s; (**e**) *t* = 1.31 × 10^−5^ s; (**f**) *t* = 1.58 × 10^−5^ s; (**g**) *t* = 1.86 ×10^−5^ s; (**h**) *t* = 2.13 × 10^−5^ s.

Brittle materials are mainly used to remove materials under the action of fracture and friction. Figure 6 shows the brittleness removal process of SiC ceramic polishing for the polishing depth *a_p_* = 1 μm, the polishing speed *V_s_* = 1151 mm/s, the apex angle of abrasive grain α = 90 °, as well as the height of the abrasive grain (*h* = 7 μm). In the initial stage, both the front and bottom of the abrasive particles start to produce tiny cracks, indicating the removal of the workpiece in a brittle mode. As shown in Figure 6b, as the abrasive moves in the cutting direction, the cutting effect eliminates the crack at the front of the abrasive grain, but is unable to eliminate that below the abrasive grains. The initiation cracks begin to gather and expand on the subsurface, forming a median crack. In this way, the SSD tends to be deepened. As shown in Figure 6c, with further cutting of the abrasive grain, a transverse crack is produced near the median crack. Owing to the squeezing and shearing effect of the abrasive particles, the stress concentration occurs on the tip and the side of the crack. The abrasive particles continue to move forward (Figure 6d), and the transverse cracks expand in the direction of the stress concentration. The stress concentration on the side of the median crack also germinates the transverse crack. When the crack is extended to a certain length, it is connected under the surface of the workpiece, resulting in the brittle removal. Figure 6e–h demonstrates that the crack undergoes three stages under the action of abrasive cutting—i.e., initiation, expansion, and cracking—to form a median crack and a transverse crack. The cracks are interconnected with the processed subsurface to form the machined surface. Meanwhile, the SSD is caused by the median crack, which is unconnected with the lateral crack.

#### 3.3.2. Effect of Polishing Depth on SSD Depth

**Figure 7:**
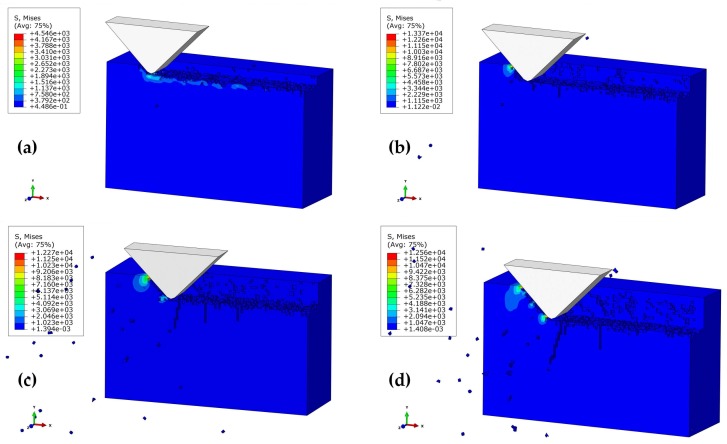
Effect of polishing depth on the depth of subsurface damage (SSD) at the polishing speed of 1361 mm/s, and the height of the abrasive grain of *h* = 7 μm: (**a**) *a_p_* = 1 μm; (**b**) *a_p_* = 2 μm; (**c**) *a_p_* = 3 μm; (**d**) *a_p_* = 4 μm.

Figure 7a–d shows the subsurface topographies of the machined workpiece at stepwise polishing depths of 1 μm, 2 μm, 3 μm, and 4 μm (*α* = 90 °, *V_s_* = 1361 mm/s, *h =* 7 μm). For *a_p_* = 1 μm, there is no observation of obvious subsurface cracks in the workpiece. *a_p_* = 2 μm further increased the contact area of the abrasive grain and the workpiece. The edge cracks resulted from the median crack, whereas the edge damage was caused by the cutting action. Figure 7c,d illustrates the obvious damage caused by median cracks at *a_p_* = 3 μm and 4 μm, while the lateral cracks caused the collapse under the effect of the cutting force. Taken together, the increase in polishing depth not only degraded the smoothness of the workpiece surface, it also destructed the subsurface and strength of the materials.

#### 3.3.3. Effect of Polishing Speed on SSD Depth

**Figure 8:**
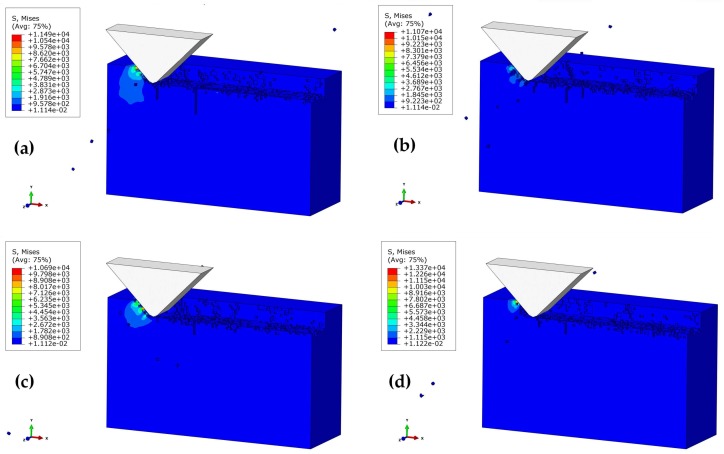
Effect of polishing speed on SSD depth at the polishing depth of 2 μm, and the height of the abrasive grain *h =* 7 μm: (**a**) *V_s_* = 523 mm/s; (**b**) *V_s_* = 733 mm/s; (**c**) *V_s_* = 1151 mm/s; (**d**) *V_s_* = 1361 mm/s.

Figure 8 illustrates the subsurface topographies of the machined workpiece at various polishing speeds (523–1361 mm/s) at the conditions of *a_p_* = 2 μm, *α* = 90°, and *h* = 7 μm. For polishing speed *V_s_* = 523 mm/s, there has been obvious subsurface cracks, and the edge of the workpiece is severely damaged. In this case, the SSD depth is approximately 4.9 μm. When the polishing speed was increased to 733 mm/s, the edge breakage tended to be weakened (SSD depth of about 3.7 μm). Further increasing the polishing speed to 1151–1361 mm/s led to the disappearance of edge breakage, and a resultant decrease in SSD. In other words, the quality of the subsurface can be improved at a relatively higher polishing speed. Herein, the polishing speed can be optimized to be 1151 mm/s, based on the above conditions.

#### 3.3.4. Effect of Abrasive Grain Size on SSD Depth

**Figure 9:**
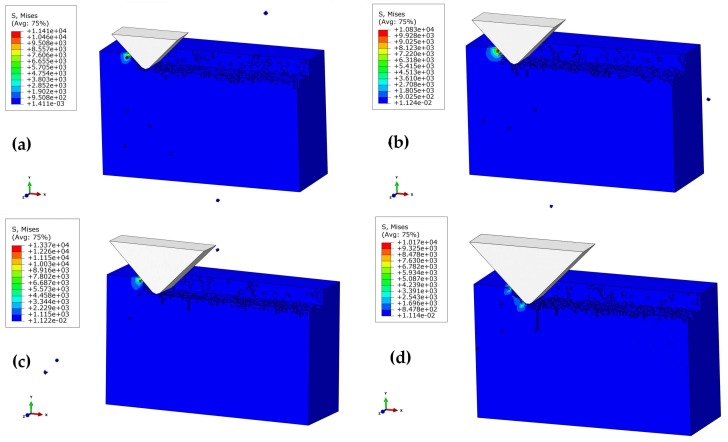
Effect of abrasive grain size on SSD depth at the polishing depth of 2 μm and *V_s_* = 1361 mm/s: (**a**) *h* = 5 μm; (**b**) *h* = 6 μm; (**c**) *h* = 7 μm; (**d**) *h* = 8 μm.

Figure 9 shows the morphologies of subsurface cracks at the abrasive height *h =* 5–8 μm. It is found that there is no obvious subsurface crack on the surface of the workpiece when the abrasive heights *h* = 5 μm, and the polishing surface is relatively smooth. There is a depth of damage caused by tiny cracks on the subsurface, and the presence of stress concentration reduced the material strength. From Figure 9a–d, it can be seen that the increase of the abrasive height will cause the propagation at the subsurface crack, and even aggravate the damage of the edge of the workpiece. Therefore, in actual processing, the small size of the abrasive can be selected to reduce the SSD.

## 4. Validation of Experimental Results

### 4.1. Experimental Preparation

**Figure 10:**
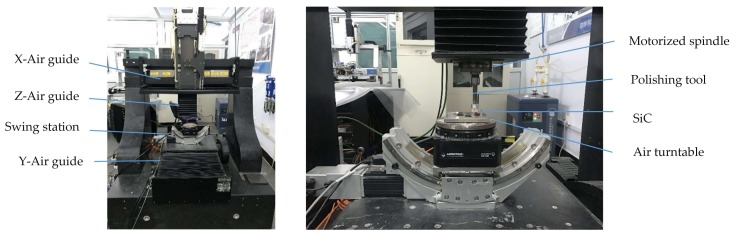
Optical photograph of experimental setups.

We performed the polishing experiments by using an in-house five-axis linkage computer-controlled precision polishing machine equipped with a diamond polishing tool (diameter of 10 mm) and polishing paste with different sizes of diamond grits (Figure 10). The rotating speed of the spindle can reach 3000 rpm, accounting for the maximum linear velocity of 1570 mm/s. The samples used here are the square-shaped pressureless sintered SiC ceramic, which is sized 10 mm × 10 mm × 2 mm.

We studied the dependence of SSD on three parameters, i.e., polishing depth (*a_p_* = 1 μm, 2 μm, 3 μm, and 4 μm), spindle speed (*V_s_ =* 1000 rpm, 1400 rpm, 2200 rpm, and 2600 rpm), and abrasive grain size (W0.5, W1.5, W2.5, and W3.5), in the polishing process. The SSD depth was determined through the observation of a cross-section microscopy. Following all of the measurements, the SiC samples were chemically eroded by HF solution (1 vol. H_2_O dest, 1 vol. HCl 38%, and 1 vol. HF 40%) to enable the visibility of subsurface cracks. After cleaning by ultrasonication several times, the cross-section images of the specimen were observed with a scanning electron microscope (SEM) (JSM-6700F, JEOL, Tokyo, Japan). Thereby, the micrographs of 10 different parts under each parameter were selected for the identification of the maximum depth of cracks (maximum depth of SSD) on a quantitative basis.

### 4.2. Experimental Results and Discussion

**Figure 11:**
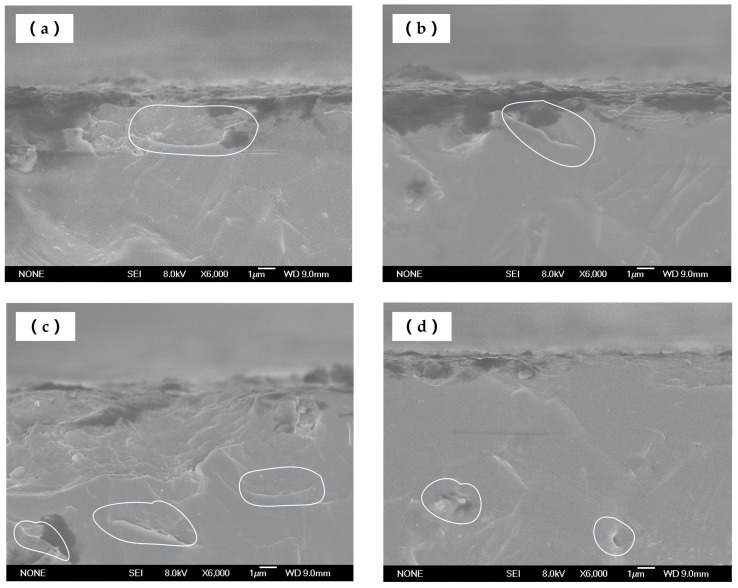
SEM images of SSD morphology of SiC for *a_p_* = 3 μm, *V_s_ =* 2200 r/min, and W2.5: (**a**) near the finished surface; (**b**) bottom of the pit; (**c**) extended surface; (**d**) far away from the surface.

Figure 11a–d show the typical SEM images of subsurface cracks beneath the ground surface at *a_p_ =* 3 μm, *V_s_* = 2200 r/min, and W2.5. Due to the coexistence of Si and the SiC phase involved in the material, the stress state would become very complex after the interaction between grains and the specimen, and thus, the direction of the cracks propagation would be random. After the propagation of these cracks, a large number of dimples were generated near the surface of the workpiece. Some cracks extended to the free surface to remove the material. As shown in Figure 11a, these pits were smooth, and other parts of the cracks continued to expand downward at the bottom of the pits. At some relatively weak points, the cracks extended to deeper locations, where the cracks were scattered. Although the interior of the material was not absolutely uniform, there were some inherent defects. In addition, the individual cracks were also found on the sites far away from the surface of the material. These should originate from the inherent defects produced within the material that were extended and driven by the polishing force (Figure 11d).

**Figure 12:**
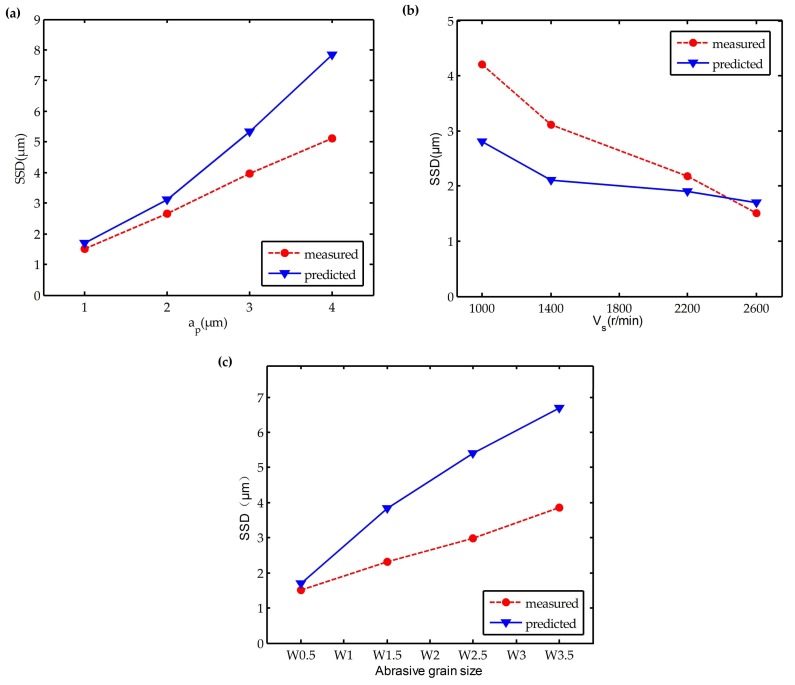
Dependence of SSD depth on polishing parameters: (**a**) polishing depth *a_p_*; (**b**) spindle speed *V_s_*; (**c**) abrasive grain size.

Substituting the process parameters in the experiment into Equation (21), the predicted values of the maximum SSD depth are obtained. As shown in the Table 5, the predicted and measured values of maximum SSD depth are listed. Figure 12 demonstrates the variations of maximum SSD depth with polishing depth *a_p_*, spindle speed *V_s_*, and an abrasive grain size that includes measured and predicted values. The SSD depth was positively correlated with an increase in the polishing depth. The most likely reason should be that the polishing depth *a_p_* and polishing force *F* of the single grit increased as the polishing depth increased. This result appeared to be in good consistence with Equation (12), i.e., increasing the polishing force eventually led to an increase in the depth of the SSD. Thereafter, the relationship between the spindle speed and the SSD depth could be obtained by a stepwise change of spindle speed. Increasing the spindle speed decreased the normal force of the single grit, which gave rise to the formation of debris, and the decrease in the plastic deformation of surface layer and the average SSD depth. However, on the other hand, the upper limitation of the spindle speed due to the spindle performance and strength of the polishing tool necessitated the consideration of the relationship between the spindle speed and the polishing tool. This determined the optimum polishing speed for improving the machining quality and processing efficiency. It is clearly visible in Figure 12c that the increased abrasive grain size worsened the SSD depth. When the abrasive grain size was increased, the actual cutting depth of the single grit was increased, which enhanced the normal contact force of the single grit and the SSD depth.

The above results and analysis clearly suggested a good agreement between the theoretical simulation and the experiments with respect to SSD depth as a function of the polishing depth, spindle speed, and abrasive grain size. However, the theoretical value was also found to be different from the experimental value. The most likely reason should be: (1) the theoretical equations are built in the static process; however, the real polishing process is a dynamic and complex situation; (2) the depth that was calculated by the theoretical equations failed to consider the problem of elastic and plastic deformation; (3) the etching time was barely controlled, which induced an etching error in the experimental tests.

## 5. Conclusions

Based on the above results, the kinematic characteristics of the polishing process and the fracture characteristics of the brittle materials are analyzed and discussed. The theoretical models were established for the description of SSD. The SSD of SiC ceramic was simulated and predicted by the finite element method. The effect of different parameters on SSD of the SiC ceramic workpiece was also analyzed, which provided a reasonable guidance for the subsequent polishing process. According to the discussion above, main conclusions can be drawn as follows:

(1) On the basis of the dynamic relationship between the abrasive particles and the workpiece in the theoretical analysis, when $h_{i}{>h}_{c}$, the material removal is mainly brittle fracture. The SSD depth increases as the abrasive grain angle and load (proportional to the polishing depth) increases, and decreases as the speed increases.
(2) In the simulation, the formation and propagation of cracks are presented. The simulation results indicate that the polishing depth, abrasive grain size, and polishing speed have the most significant effect on SSD, respectively. By increasing polishing depth and abrasive grain size, the SSD depth increases, and an increasing polishing speed results in a decrease in SSD depth.
(3) The polishing experiments under different processing parameters are carried out. The experimental results validate the theoretical and simulation results.

## Figures and Tables

**Table 1:** Values of $d_{m}$, $d_{\max}$ and *σ* for different grit designations.

*M* (Mesh Size)	$d_{\max}$ (μm)	$d_{m}$ (μm)	*σ* (μm)
180	84.4	47.3	12.4
320	47.5	21.1	8.8
400	38.0	15.5	7.5
600	25.3	8.8	5.5
800	19.0	5.9	4.4
1000	15.2	4.3	3.6
1500	10.1	2.4	2.6
2000	7.6	1.6	2.0
4000	3.8	0.6	1.1
6000	2.5	0.35	0.7
10,000	1.5	0.17	0.4

**Table 2:** The physical parameters of silicon carbide (SiC) material.

Material Properties Parameters	Value
Density *ρ* (kg/m^3^)	3215
Elastic modulus *E* (GPa)	454
Poisson’s ratio	0.25
Yield strength *σ* (MPa)	620
Specific heat [J/(kg·K)]	526.3
Conductivity [W/(m·K)]	180
Hardness (GPa)	29.4

**Table 3:** The model constants of SiC [13].

**Constitutive Model**	$\rho_{0}\left(\mathrm{kg}/m^{3}\right)$	G(GPa)	***A***	***N***	***B***	***M***	***C***
	**3215**	**193**	**0.96**	**0.65**	**0.35**	**1.0**	**0.009**
	$\sigma_{\max}^{i}\left(\mathrm{GPa}\right)$	$\sigma_{\max}^{f}\left(\mathrm{GPa}\right)$	HEL(GPa)	$P_{\mathrm{HEL}}\left(\mathrm{GPa}\right)$	***β***	${\overset{\cdot }{\varepsilon }}_{0}$	T(GPa)
	**12.2**	**1.3**	**11.7**	**5.13**	**1.0**	**1.0**	**0.75**
	*β*	$K_{1}\left(\mathrm{GPa}\right)$	$K_{2}\left(\mathrm{GPa}\right)$	$K_{3}\left(\mathrm{GPa}\right)$			
	1.0	220	361	0			
**Failure Model**	$D_{1}$	$D_{2}$	${\bar{\varepsilon }}_{f,\max}^{pl}$	${\bar{\varepsilon }}_{f,\min}^{pl}$	*FS*	*Damage*	
	0.48	0.48	1.2	0.0	0.2	0	

**Table 4:** Parameters for numerical simulation.

Polishing Parameters	Value
Polishing depth *a_p_* (μm)	1, 2, 3, 4
Polishing speed *V_s_* (mm/s)	523, 733, 1151, 1364
Abrasive grain size *h* (μm)	5, 6, 7, 8

**Table 5:** The predicted and measured values of maximum SSD depth.

Test	Polishing Depth *a_p_* (μm)	Polishing Speed *V_s_* (mm/s)	Abrasive Grain Size	Predicted SSD (μm)	Measured SSD (μm)
1	1	2600	W 0.5	1.697	1.515
2	2	2600	W 0.5	3.110	2.231
3	3	2600	W 0.5	5.340	3.961
4	4	2600	W 0.5	7.834	5.113
5	1	1000	W 0.5	2.804	4.212
6	1	1400	W 0.5	2.102	3.116
7	1	2200	W 0.5	1.898	2.176
9	1	2600	W 1.5	3.844	2.325
10	1	2600	W 2.5	5.398	2.987
11	1	2600	W 3.5	6.697	3.857
